# Adsorption of Hg^2+^/Cr^6+^ by metal-binding proteins heterologously expressed in *Escherichia coli*

**DOI:** 10.1186/s12896-024-00842-9

**Published:** 2024-03-23

**Authors:** Shuting Hu, Zixiang Wei, Teng Liu, Xinyu Zuo, Xiaoqiang Jia

**Affiliations:** 1 Department of Biochemical Engineering, School of Chemical Engineering and Technology, Tianjin University, Tianjin, 300072 PR China; 2 Frontier Science Center for Synthetic Biology and Key Laboratory of Systems Bioengineering (MOE), School of Chemical Engineering and Technology, Tianjin University, Tianjin, 300350 PR China

**Keywords:** Engineered *E. coli*, Bioremediation, Heavy metals, Detoxification, Transcription protein

## Abstract

**Background:**

Removal of heavy metals from water and soil is a pressing challenge in environmental engineering, and biosorption by microorganisms is considered as one of the most cost-effective methods. In this study, the metal-binding proteins MerR and ChrB derived from *Cupriavidus metallidurans* were separately expressed in *Escherichia coli* BL21 to construct adsorption strains. To improve the adsorption performance, surface display and codon optimization were carried out.

**Results:**

In this study, we constructed 24 adsorption engineering strains for Hg^2+^ and Cr^6+^, utilizing different strategies. Among these engineering strains, the M’-002 and B-008 had the strongest heavy metal ion absorption ability. The M’-002 used the flexible linker and INPN to display the *merR*_*opt*_ at the surface of the *E. coli* BL21, whose maximal adsorption capacity reached 658.40 μmol/g cell dry weight under concentrations of 300 μM Hg^2+^. And the B-008 overexpressed the *chrB* in the intracellular, its maximal capacity was 46.84 μmol/g cell dry weight under concentrations 500 μM Cr^6+^. While in the case of mixed ions solution (including Pb^2+^, Cd^2+^, Cr^6+^ and Hg^2+^), the total amount of ions adsorbed by M’-002 and B-008 showed an increase of up to 1.14- and 4.09-folds, compared to the capacities in the single ion solution.

**Conclusion:**

The construction and optimization of heavy metal adsorption strains were carried out in this work. A comparison of the adsorption behavior between single bacteria and mixed bacteria systems was investigated in both a single ion and a mixed ion environment. The Hg^2+^ absorption capacity is reached the highest reported to date with the engineered strain M’-002, which displayed the *merR*_*opt*_ at the surface of chassis cell, indicating the strain’s potential for its application in practical environments.

**Supplementary Information:**

The online version contains supplementary material available at 10.1186/s12896-024-00842-9.

## Introduction

Heavy metals are naturally occurring elements in the earth’s crust that are rare in the biological environment. However, their industrial, domestic, agricultural, medical, and technological applications have led to widespread heavy metal pollution in human habitats, seriously threatening health and the environment [1, 2]. Their toxicity is dependent on the dose, exposure route, and chemical species. The highly toxic elements such as mercury (Hg) and chromium (Cr) are moreover considered systemic toxicants [1], and exposure to these metals through contaminated food or water can cause damage to multiple organs or cause cancer even at low intake levels [3]. Due to the harm caused by the widespread distribution of heavy metal ions, there has been a long-standing focus on their removal. The main process and arguably the most efficient process for heavy metal removal is chemical precipitation [4, 5], where heavy metals are precipitated as insoluble complexes and bound in particles [6]. In addition, physical methods such as adsorption [7], solvent extraction [8], ion exchange [9], and membrane separation [10] can also be used for the separation and purification of heavy metals. However, all these methods have drawbacks, such as high equipment and material costs, high energy requirements, and the risk of secondary contamination [11, 12].

In the last decades, the potential of biosorption of metals has been increasingly explored [13]. High metal-adsorbing biomass provides a basis for newly developed metal bioremediation processes such as bioflocculation, phytoremediation, and biosorption [14–16], especially as a highly competitive means of detoxifying metal-containing industrial wastewaters [17]. Recently, removing heavy metals by microbes is becoming a potentially exploitable method, because of its security, high efficiency, and practicability, with the major advantages of no/reduced accumulation of secondary pollutants, cost-effectiveness, and high metal recovery [18]. Bacteria, fungi, and microalgae were used to realize Hg^2+^ or Cr^6+^ removal achieving different adsorption capabilities (shown in Table 1). Most of the studies used natural microorganisms screened in heavy metal-rich environments, and used them directly, immobilized them, or modified them by physical/chemical methods for adsorption. Biosorption capacity was commonly used to evaluate the performance of strains, but the removal rate was not presented in some articles. The complexity of undomesticated microorganisms poses limitations for further strain optimization, so it is needed to deeply understand the adsorption principles of microbial cells to enable targeted enhancements based on this knowledge [19].

**Table 1:** Biosorption by microbes

Metal ions	Species	Biosorption capacity (μmol/g)	Removal rate	Source or form of biosorbents	References
Hg	*Penicillium canescens*	273.2		Free cells	[20]
Hg	*Penicillium purpurogenum*	351.0		Free cells	[21]
Hg	*Escherichia coli* BL21		43.7%	Free cells	[22]
Hg	*Bacillus cereusb*	519.0		immobilized cells	[23]
Hg	*Bacillus sp*	39.6		Free cells	[24]
Hg	*Escherichia coli* BL21	658.66	93.0%	Heterologous protein expression	Our study
Cr	*Saccharomyces cerevisiae*	154.8	99.6%	Chemical and thermal treatments	[25]
Cr	*Sargassum oligocystum*	662.7		CaCl_2_-modified	[26]
Cr	*Sphingopyxis macrogoltabida* SUK2c	38.5	55%	Free cells	[27]
Cr	*Bacillus licheniformis*	1334.6		dead cells	[28]
Cr	*Cronobacter muytjensii* KSCAS2		76.51%	Free cells	[29]
Cr	*Acinetobacter junii* VITSUKMW2	426.9		Free cells	[30]
Cr	*Escherichia coli* BL21	46.73	24.2%	Heterologous protein expression	Our study

Various microorganisms have evolved resistance mechanisms to survive exposure to heavy metal ions in the environment [31]. Operons that confer resistance to heavy metals in bacteria encode proteins involved in sensing, transport, and detoxification. Transcriptional factors encoded by the operons are heavy metal-binding proteins (HMBP) that can coordinate and bind functional groups to heavy metal ions through conformational changes.

The *mer* and *chr* operons confer resistance to mercury and chromium, respectively. The *mer* operon is widely distributed in bacteria and has a complex composition [32, 33]. MerC, MerP, and MerT are the key components for the recognition and uptake of Hg^2+^. MerA and MerB catalyze the reduction of organic mercury or Hg^2+^ into less toxic valence states [34], while the transcriptional factor MerR coordinates the expression of this operon [35]. The core Hg(II)-binding domain of the MerR dimer is constituted by a pair of antiparallel α-helices with 3 cysteine residues (Cys38, Cys117 and Cys126) of each monomer [36, 37]. High affinity of Hg(II) to SH residues allows the generation of trigonal planar coordination between Hg(II) and cysteine residues [38]. The favorable tertiary interactions in protein systems such as merR go a long way in stabilizing nonnatural coordination environments in biological systems [39].

The *chr* operon, including *chrB*, *chrA*, *chrC,* and *chrF*, is derived from the highly Cr(VI)-resistant bacterial strain *Ochrobactrum tritici* 5bvl1 isolated from chromium-contaminated wastewater [40–42]. As a chromium-responsive HMBP, ChrB has the functions of sensing Cr(VI) and regulating the transcription of the *chr* operon [43]. Currently, research into the specific mechanisms responsible for the activity of the Chr transcription regulatory protein family remain limited. ChrB also dimercally binds with Cr(VI). Branco et. al. found that the three amino acids (R180, R187 and H229) might play a critical role in the process of Cr(VI) induction, which appear to be part of the Cr(VI) binding site within the ChrB protein [43].

For the vast majority of natural microorganisms, the stress of heavy metal ions is lethal but uncommon, and only in rare cases do microorganisms encounter environments with a high concentration of heavy metal contaminants that activate resistance mechanisms [44]. Thus, the metabolism of natural microorganisms is not adapted to the adsorption of contaminated wastewater, and even if they are able to catalyze some adsorption, further modification, such as overexpression of HMBPs, is required to take advantage of the potential of microbial adsorption. *Cupriavidus metallidurans* CH34 was a model bacterium to study bacterial resistance to metals, and its genome sequence analysis revealed the presence of a variety of paralogs of proteins that were previously shown to be involved in heavy metal resistance [45]. We have gained a lot of insight into the principles of bacterial heavy metal resistance by researching this strain, so we chose operons from this strain for further investigation [46]. Surface display is a recombinant technology that expresses target proteins on cell membranes, and this technique has been used for various biotechnical and biomedical applications such as drug screening, biocatalysts, library screening, quantitative assays, and biosensors [47]. It is also an effective avoidance of substance transfer limitations and protein instability [45], by transporting various affinity proteins out of cells and fusing them with anchoring proteins to immobilize onto the cell surface as displayed proteins. Because of this characteristic, surface display technology has been used to modify natural microorganisms for heavy metal adsorption.

In this study, we used MerR and ChrB that derived from *Cupriavidus metallidurans* CH34, applied the anchoring motif INPN to express the HMBPs on the outer surface of the cell membrane and overexpressed the HMBPs to construct engineered strains capable of efficiently adsorbing heavy metal ions. Because we noticed that, due to the complexity of the actual polluted environment, the Hg^2+^ in the pollutants would seriously affect the adsorption effect during the biosorption process of Cr^6+^, we constructed the adsorption engineered strains of these two kinds of heavy metal pollution at the same time, and intended to utilize the engineered strains to achieve heavy metal adsorption and removal in the environment [48]. Then, their adsorption performance was evaluated by quantifying the adsorption capacity and adsorption rate, calculated based on the dry weight of the bacteria adsorbent and the metal content quantified after microwave digestion and impurities removal. As a result, the highest mercury adsorption reported as date was obtained by our engineered strain M’-002 under 300 μM Hg^2+^ solution. However, the adsorption of Cr^6+^by the engineered strain B-008 was unsatisfactory, only 46.84 μmol/g cell dry weight under concentrations 500 μM Cr^6+^ and there was still much space for optimization. Then, we have proved that the adsorption of the engineered strains in different environments is workable by investigating and comparing the adsorption behavior in the presence of either single ion or mixed ions. Studies on the possible synergistic influences on the adsorption performance of heavy metals were also conducted, and clearly demonstrated a notable difference to the adsorption in mixed bacteria systems. With this study we demonstrated the feasibility of utilizing HMBPs from natural microorganisms to construct engineered adsorbent strains by the use of genetic techniques, and provided the tools for achieving bioremediation for actual environments.

## Materials and methods

### Strains, plasmids, and culture conditions

*E. coli* BL21(DE3) was used as the host for the designed adsorption bacteria. *E. coli* BL21(DE3) was purchased from TransGen Biotech Co., Ltd. Cells were grown aerobically in Luria–Bertani broth (LB) containing 5 g/L yeast extract, 10 g/L sodium chloride, and 10 g/L peptones with an adjusted pH of 7.2–7.4. The plasmid pET28a was used as the vector for cloning and protein expression. Unless otherwise noted, all bacterial strains were cultured at 37 °C to the logarithmic growth phase (optical density [OD_600_] = 0.6–0.8) and then cooled down to 22 °C for protein expression.

### Plasmid construction

The HMBP overexpression strains B-008 and M’-006 were construsted by simply inserting transcription factors (TFs) genes into pET28a plasmid. TFs were amplified by polymerase chain reaction (PCR), and the amplification templates of gene *chrB* (GenBank: CP000355.2) and *merR* (GenBank: CP050332.1) were the plasmid pMOL28 from *Cupriavidus metallidurans* (ATCC 43123D-5). *EcoR*I and *Hind*III sites were added for primers of TFs. The TFs coding sequences were digested with corresponding restriction endonuclease, and introduced on the pET28a plasmid by T4 DNA ligase.

Engineered plasmids of extracellular adsorption strains, B-001 ~ B-005 and M-001 ~ M-005, were constructed first by PCR amplification of the carrier proteins (INPNs) and TFs. INPN (GenBank: CP050332.1) was coded by the *P. syringae* gene *inaK* (Accession no. NC AF013159) as an anchor (shown in Table 2). Four different linker peptides were used: flexible linker (FL), rigid linker (RL), rigid helical linker(HL), or 96 bp intermediate repeating sequence [49]. 5 laboratory conservation plasmids pE-NL, pE-FL, pE-RL, pE-HL, and pKE-FL provided the linker and the amplification templates for INPN. To fuse the INPN and TFs on plasmids, *Hin*dIII and *Nhe*I recognition sites were added up- and downstream of the primers of linker, while *Nhe*I and *Xho*I sites were added for primers of TFs. The amplified INPNs and TFs were also linked to the linker corresponding plasmid via restriction enzymes and T4 DNA ligase.

**Table 2:** Plasmids used in this study

Plasmid	Assembly order	Plasmid	Assembly order
M-001	INPN-MerR	B-001	INPN-ChrB
M-002	FL-MerR	B-002	FL-ChrB
M-003	HL-MerR	B-003	HL-ChrB
M-004	RL-MerR	B-004	RL-ChrB
M-005	96 bp-MerR	B-005	96 bp-ChrB
M’-001	INPN-MerR_opt_	B’-001	INPN-ChrB_opt_
M’-002	FL-MerR_opt_	B’-002	FL-ChrB_opt_
M’-003	HL-MerR_opt_	B’-003	HL-ChrB_opt_
M’-004	RL- MerR_opt_	B’-004	RL- ChrB_opt_
M’-005	96 bp- MerR_opt_	B’-005	96 bp- ChrB_opt_
M-006	E-MerR-H	B-008	B-ChrB-E
M’-006	E -MerR_opt_-H	B’-008	B- ChrB_opt_-E

*E* EcoRI, *H* HindIII, *B* BamHI

Codon optimized plasmids were obtained by replacing TFs into optimized genes. The codon optimized genes *chrB*_*opt*_ or *merR*_*opt*_ were synthesized by GENEWIZ Co., Ltd. As previously described, 24 plasmids were constructed and transformed into BL21(DE3). All restriction enzymes, plasmid extraction kits, PCR product purification kits, and agarose gel recovery kits were purchased from TransGen Biotech Co., Ltd.

### Cell growth and protein expression

We conducted PCR verification to screen plasmids in BL21(DE3), preserved the bacteria with correct sequences in glycerol, and pre-screened the engineered strains based on adsorption capacity. Single colonies of each strain containing the target plasmid were picked and precultured overnight in 5‐ml of LB medium containing kanamycin at 37℃ in a shaker to revive the bacteria. These mixtures were then diluted with LB medium to obtain an OD_600_ value of 1. Then, 100 μL of the dilution were transferred to a shake flask containing 100 ml of medium without antibiotics for a subculture to an OD_600_ of 0.6 to 0.8. Once the cells reached the logarithmic growth phase, the inducer isopropyl‐β‐D‐thiogalactopyranoside (IPTG) was added to each flask to a final concentration of 0.2 mM. After 12 h of protein expression at 22 °C, the cultures were centrifuged at 6,000 rpm for 5 min to separate cell pellets and culture supernatant for subsequent experimental analysis. All experiments were performed in triplicates. *E. coli* BL21(DE3) without plasmids and the same strain with the empty vector pET28a were included as negative controls.

### Metal adsorption by engineered bacteria

To identify appropriate metal-binding proteins for further research, 10 engineered *E. coli* containing the above-listed plasmids were pre-screened to test their adsorption capability for corresponding heavy metals (Hg and Cr) and compared with the wild-type BL21. The strains were cultured to the logarithmic growth phase with an OD_600_ of 0.6–0.8. Then, the inducer IPTG was added to a final concentration of 0.2 mM, and corresponding ions were added to a final concentration of 200 μM. After culturing at 22 °C and 220 rpm for 12 h, the cells were collected, washed with ddH_2_O three times, dried in an oven at 60 °C for 24 h, and weighed. After microwave digestion, the heavy metal ions content in samples were respectively determined by atomic absorption spectroscopy (AAS). The adsorption capacity (μmol/g CDW (cell dry weight)) was evaluated by calculating the ratio of total metal content (μmol) to the dry weight of the bacteria (g). The effects of the protein fusion arrangement on the adsorption were also studied analogously.

The growth curves of engineered strains were recorded by measuring the OD_600_ every 2 h. The optimal induction condition was determined by studying the changes of adsorption capacity under different IPTG concentrations (0.2 mM, 0.4 mM, 0.6 mM, 0.8 mM, 1.0 mM, or 1.2 mM), temperature (16℃, 18℃, 20℃, 22℃, 24℃, 26℃, or 28℃) and time (12 h, 14 h, 16 h, 18 h, 20 h, 22 h, or 24 h). All experiments were controlled variable experiments carried out under the optimal conditions for other variables. And all experiments were designed with three parallel groups.

This study also investigated the adsorption capacity of the engineered bacteria at different heavy metal (Cr^6+^ or Hg^2+^) concentrations (50, 100, 300, 500, 1,000, or 1,500 μM) as well as the adsorption selectivity in the presence of mixed metal ions with another two typical toxic elements Cd^2+^ and As^3+^. In the mixed heavy metal ion adsorption experiments, the concentrations of the four heavy metal ions were 40, 100 and 200 μM, respectively. And in the mixed metal ions experiments, the engineering strains were separately activated and co-cultured in the fresh LB medium at an inoculation ratio of 1% (v/v). The adsorption capacity of a mixed bacterial system constructed by co-culturing the optimal strains were also investigated. Similarly, the experiments were designed with three parallel groups.

## Results

### Construction of strains

**Fig. 1:**
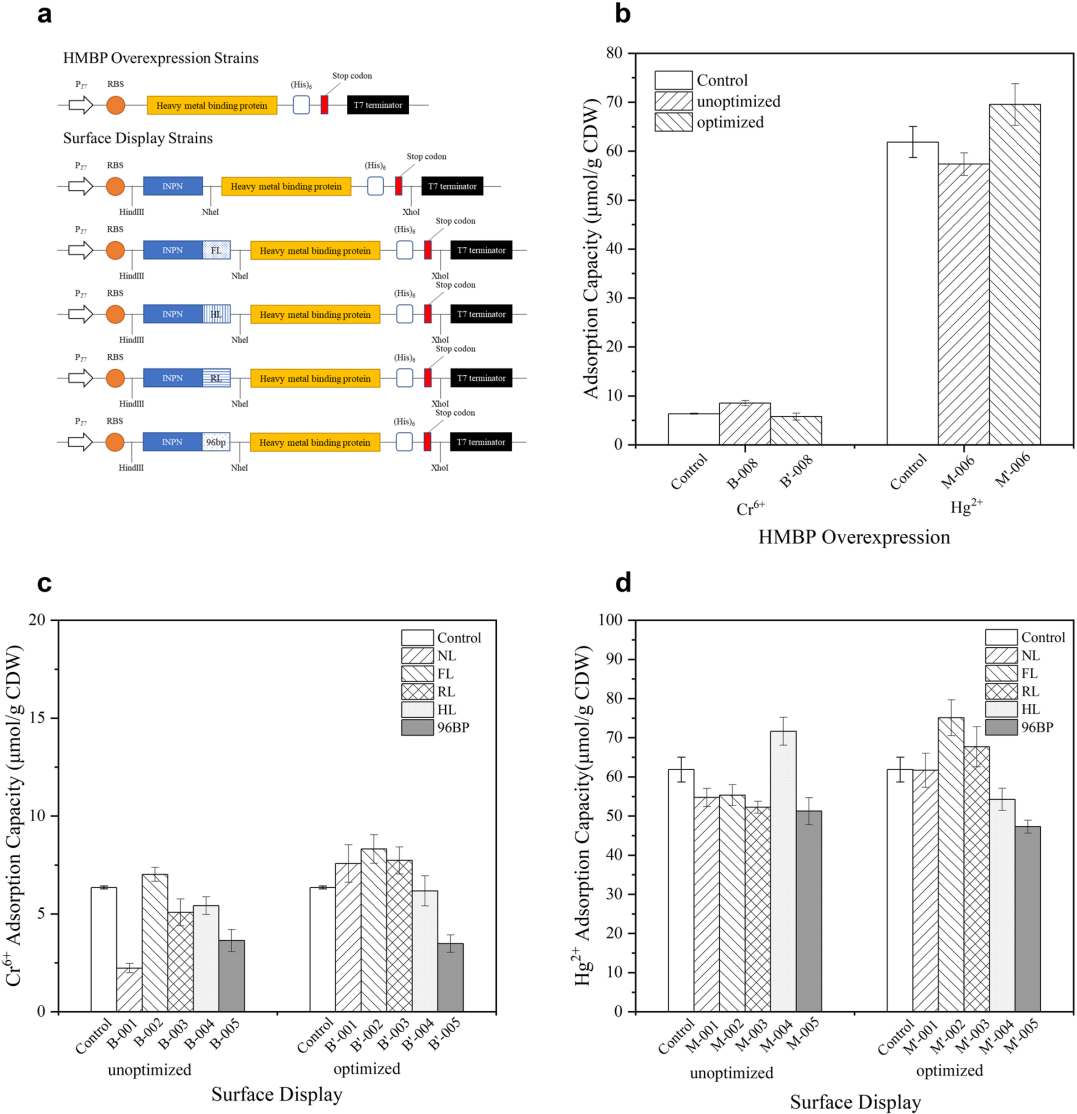
Construction and pre-selection of adsorption strains. **a** The profile of the artificial plasmids. **b**-**d** The influence of different elements and optimization methods on the ability of engineered strains to adsorb heavy metals. All data shown are the mean ± SEM of three independent experiments

We constructed two types of strains, with either intracellular protein expression or extracellular protein display, differed in adsorption site. The strategies used for cellular adsorption is summarized in Fig. 1a. In each strain, one of the HMBP genes *merR*, *merR*_*opt*_, *chrB,* or *chrB*_*opt*_ is inserted in the plasmid pET28a, and under the control of the strong T7 promoter. The T7 promoter was chosen for its powerful functionality, specificity, and controllability, with no additional metabolic burden on the cells during growth and rapid expression of the adsorbed protein after induction with IPTG. Our study linked the HMBP genes with the anchoring motif INPN and integrated them into the plasmid pET28a. Five linkers between HMBP and INPN were designed to ensure the correct conformation of the target protein in the fusion state: (a) no linker (NL); (b) an FL peptide composed of four glycines and one serine (GGGGS); (c) an RL peptide rich in proline (PAPAP); (d) a rigid HL peptide (AEAAAKEAAAKA); and (e) a 96 bp repeat region. The stop codon of the INPN fragment was removed, and a (His)6 tag was introduced before the stop codon of the HMBP [49]. A total of 24 engineered strains expressing the HMBP overexpression and surface display adsorption systems were obtained after selection and identification by colony PCR (Figure S1).

### Comparison and selection of optimal strains

We tested optimal growth condition and adsorption capability of a series of strains expressing the specific HMBPs, determining the appropriate linker for surface-displaying strains. We also studied the affection of codon optimization and both expression strategies of intracellular and extracellular based on their performance. The most effective strain was then identified and applied to subsequent adsorption experiments.

#### Growth curve analysis

Growth curves can be used to assess whether cell growth is inhibited by recombinant genes. As shown in Figure S2, the growth curve of the surface display strains and the HMBP overexpression strains basically matched the wild-type BL21, indicating that the plasmid transferred into BL21 had no obvious effect on the cell growth. The engineered strains all entered the logarithmic phase after 2 h of culture and entered the stable phase after 16 h.

#### Pre-screening

Although the two implemented proteins, MerR and ChrB, have demonstrated excellent Hg^2+^ or Cr^6+^ binding properties in previous studies [50], they have not been applied and evaluated for bioremediation using cell surface display. In the process of surface display, the choice of linker may affect the structure and function of the HMBPs, which is the key to efficient extracellular adsorption. Therefore, we measured the metal ion adsorption capacity per CDW of the strains displaying either of two proteins fused with the anchor using NL, FL, RL, HL, or 96 bps. Heterologous proteins *merR* and *chrB* are probably limited in expression in *E. coli*. Codon optimization is a process used to improve gene expression and increase the translational efficiency of a gene of interest. We optimized the HMBP coding sequences to accommodate codon bias of the host organism, resulting in *merR*_*opt*_ and *chrB*_*opt*_. Displacing the codon optimazed HMBPs in all curcuits for HMBP overexpression and surface display adsorption, the performance of 12 recombinant strains for intracellular adsorption was first assayed.

As shown in Fig. 1b-d, among the five fusion methods, the flexible linker (G4S) resulted in the highest extracellular adsorption capacity of Cr^6+^ and Hg^2+^ in the corresponding strains B’-002 and M’-002. This may be related to the flexibility and stability of FL, which allows the target protein to keep the optimal folded structure and maximal biological activity while maintaining a certain distance from the anchor protein [49]. While the codon optimization did not show an obvious advantage in intracellular adsorption. In fact, the average Cr^6+^ adsorption capacity of non-optimized *chrB* was higher than that of the codon-optimized strain. Among the engineered strains, B-008 and M’-006 exhibited the strongest intracellular adsorption of Cr^6+^ and Hg^2+^, respectively. Besides, some engineered strains showed a decrease in adsorption capacity after codon optimization, the possible reason was that the unsuitable induction conditions led to an increase in formation of inclusion body. Therefore, the experiments to optimize the induction conditions were operated.

### Factors influencing heavy metal adsorption

Inducer concentration, temperature, and induction time may affect the expression effect of HMBPs; therefore, we designed one-way experiments for these three factors to determine the optimal induction conditions for the target strains, using adsorption capacity as an indicator.

#### Inducer concentration

**Fig. 2:**
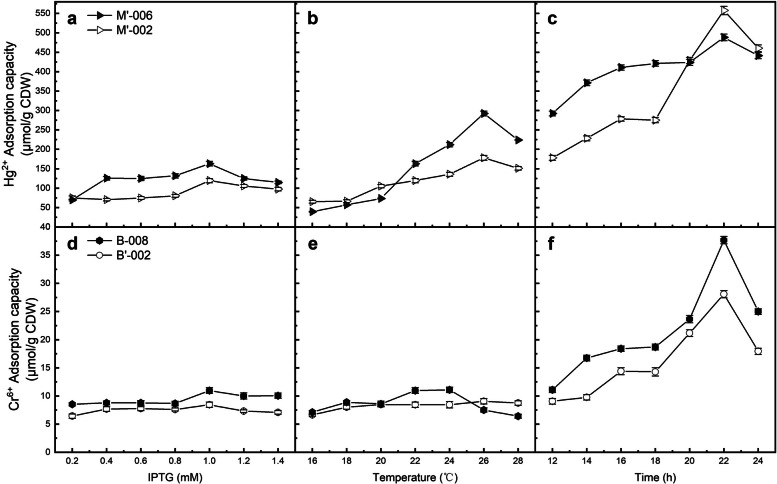
Effect of induction conditions on the heavy metal adsorption capacity of the engineered bacteria. **a**-**c** Mercury adsorption strains; **d**-**e** Chromium adsorption strains. All data shown are the mean ± SEM of three independent experiments

We tested the adsorption capacity of the engineered strains at IPTG concentrations of 0.2 ~ 1.4 mM. As shown in Fig. 2a and d, the adsorption capacity of all strains demonstrated a peak at an IPTG concentration of 1.0 mM. When the IPTG concentration was in the range of 0.4 mM ~ 1.0 mM, the adsorption capacity escalated slightly with the increasing inducer concentration. Once the IPTG concentration was up to 1.0 mM, the engineered strains peaked the adsorption capacity and then dropped as the further increasing IPTG concentration. Keeping IPTG concentration appropriate is necessary to maintain a maximum HMBP concentration within overexpression limits. Since the HMBPs bind metals in proportion of concentration, the adsorption capacity is expected to step with increasing soluble protein expression levels. However, excessive overexpression can lead to cellular stress, and also result protein misfolding and the aggregation of inclusion bodies, which may undermine the function of the HMBPs [49]. Moreover, the expression of heterologous proteins often reaches an upper limit due to the finite recourses in the cell. Therefore, an optimal inducer concentration is expected to give the maximum adsorption capacity, which reaches a balance among induction efficiency, toxicity, and overexpression. In this study, 1.0 mM IPTG was selected as the optimal inducer concentration for subsequent experiments.

#### Temperature and time

As shown in Fig. 2b and e, when the induction temperature raised up, all curves peaked and then falled, which was more obvious for the mercury adsorption strains. 26 °C is the most optimal adsorption condition for the three strains, M’-002, M’-006, and B’-002, while the adsorption capacity of B-008 reached the maximum at 24 °C. Additionally, the curves also indicate that the extracellular adsorption is relatively stable, whereas the HMBP overexpression strains were more temperature-sensitive. We speculate that the surface display provides an immobilized environment for HMBPs, so it can perform stably under changed environmental conditions. In this study, 26℃ was selected for M’-002, M’-006, and B’-002, while 24℃ was selected as the optimal expression temperature for B-008. And in this study, the best induction time of the four engineered strains was 22 h, as shown in Fig. 2c and f. The result indicated that excess induction time is also negative to adsorption.

#### Adsorption kinetics analysis

Following the methodology of Li et al. and Lu et al., we utilized the pseudo-first-order and pseudo-second-order kinetic models to identify the adsorption kinetic models of the four strains based on the time- adsorption capacity relationship. The expression of the models is generally described in the following equation:

- The pseudo-first-order equation: $\ln \left( {q_{e} - q_{t} } \right) = \ln q_{e} - K_{1} t$.
- The pseudo-second-order equation: $\frac{t}{{q_{t} }} = \frac{1}{{(K_{2} q_{e}^{2} )}} + \frac{t}{{q_{e} }}$.

Where $q_{e,cal}$ and $q_{t}$ are the adsorption capacity (mg/g) of heavy metal ions on the biomass at equilibrium and at time t, respectively. $K_{1}$ is the first-order rate constant (min^−1^), and the $K_{2}$ is the rate constant of pseudo-second-order sorption (g mg^−1^ min^−1^). The $q_{e,cal}$, $K_{1}$, and $K_{2}$ are calculated from the intercepts and the slopes of the straight lines.

We analyzed the data and obtained the pseudo-first-order results for the adsorption process (as shown in Table 3), and the adsorption process did not conform to the pseudo-second-order equation according to the calculation. Although the fitting of our model was weak and the values of $q_{e,cal}$ varied considerably from the $q_{e,\exp }$, the value of $K_{1}$ could explain our experimental phenomena to some extent. The value of $K_{1}$ for M’-006 and -008 was lower, and the adsorption processes of M’-006 and B-008 took longer to reach the equilibrium period correspondingly.

**Table 3:** Adsorption kinetics analysis

**Strains**	**Pseudo-first-order model parameters**			***q***_***e***_ (mg/g)
	***q***_***e cal***_ (mg/g)	***K***_***1***_ (min^−1^)	***R***^***2***^	
M’-002	336.6	0.1157	0.7624	111.88
M’-006	177.0	0.1394	0.8786	97.96
B’-002	4.455	0.1153	0.8083	1.46
B-008	3.057	0.0688	0.9001	1.96

### Effects of heavy metal concentration and species on adsorption

#### Metal-concentration-depending adsorption capacity

Maximal adsorption capacity is the core attribute of heavy metal adsorption strains, defined by the ratio of metal content (μmol) to the biomass (g CDW), characterizing the ability of the bacteria to adsorb metal ions. Adsorption rate is also an important parameter, which represents the metal ions adsorbed by the bacteria in proportion to the total ions in solution [51]. It has been reported that the biosorption of heavy metals is affected by the ion concentration in the environment. To survey the relationship between the ion concentration and the adsorption performance, these engineered strains, including the wild-type BL21, were incubated in LB medium containing different concentrations of Hg^2+^ or Cr^6+^ (50–1,000 μM) for 12 h.

**Fig. 3:**
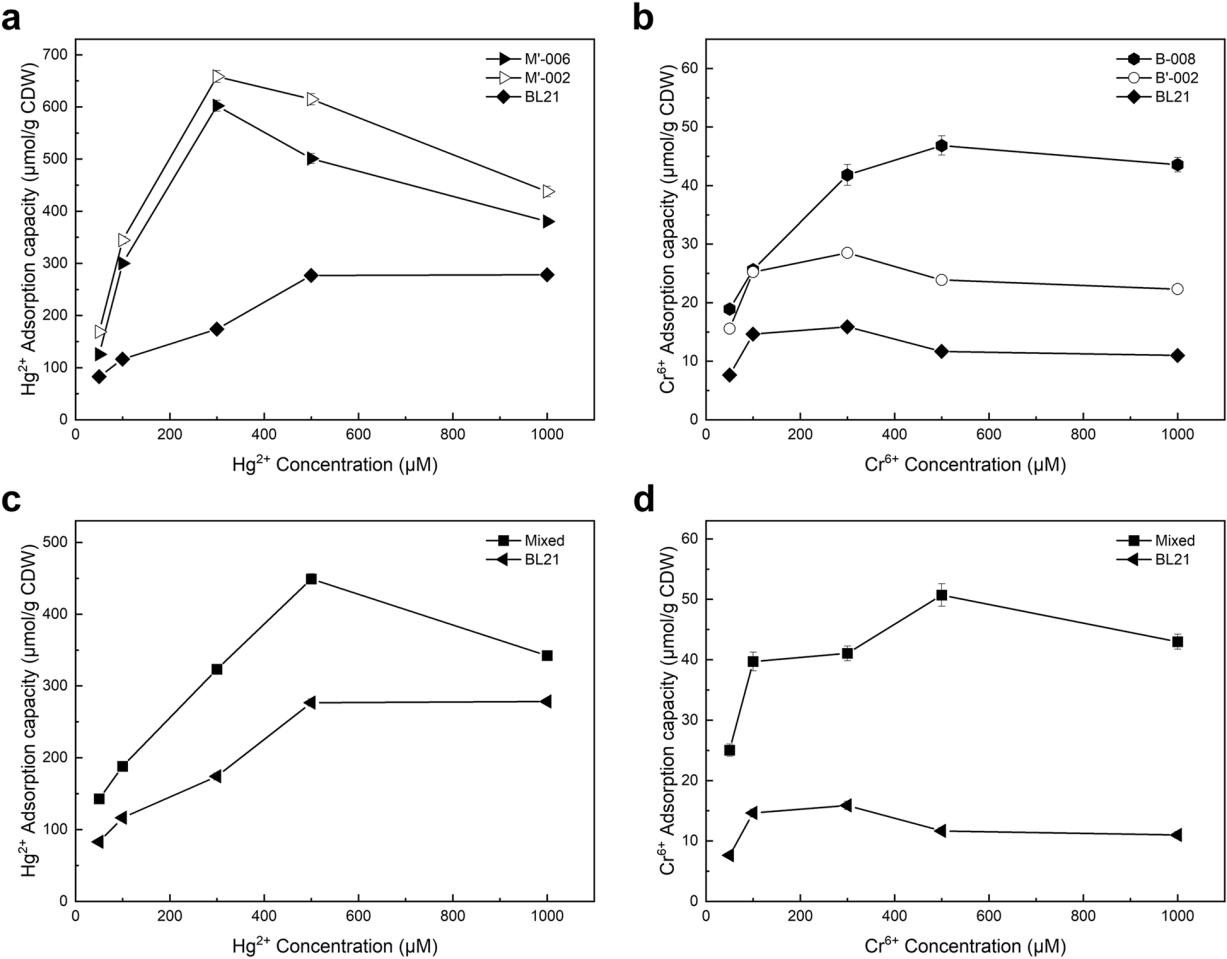
Effect of Hg^2+^ or Cr^6+^ concentrations on the adsorption capacity of engineered strains. The colored lines indicate engineered strains, and the black lines indicate the wild-type BL21. **a** Mercury adsorption strains; **b** Chromium adsorption strains; **c** and **d** Mixture of strains M’-002 and B-008. All data shown are the mean ± SEM of three independent experiments

The effect of the Hg^2+^ and Cr^6+^ concentrations on the adsorption capacity of the engineered bacteria is shown in Fig. 3a and b. The Hg^2+^ and Cr^6+^ adsorption capacity of all strains performed peaking and then falling. As the concentration of Hg^2+^ increased from 0 to 300 μM, the adsorption capacity of M’-002 and M’-006 spiked to 658.66 and 602.34 μmol/g CDW and then gradually decreased as the concentration further went up. The adsorption capacity of chromium adsorption strains B’-002 and B-008 exhibited a much smoother trend consists with an initial fast upward trend, followed by slow growth to the apex, and finally, a steady decline, which reached the maximum of 28.46 and 46.73 μmol/g CDW at Cr^6+^ concentrations of 300 and 500 μM, respectively. The distance between maximum adsorption indicted that MerR has a much higher metal affinity than ChrB, differing in their intrinsic features. MerR, therefore, showed a much stronger capacity for the binding of heavy metal ions than ChrB. This may be derived from the differ between Hg and Cr toxicity to bacteria, which created contrasting selection pressures during the evolution of the two HMBPs showing the limitations of nature proteins.

The dry weights of the adsorption strains and wild-type BL21 were measured as shown in Table S2. B’-002 and B-008 cells attained higher cell dry weights than M’-002 and M’-006 within the same metal concentration. This suggests that Hg^2+^ was significantly more toxic to *E. coli* than Cr^6+^ at the same molar concentration, which is in good agreement with previous studies [52].

#### Adsorption of mixed ions

**Fig. 4:**
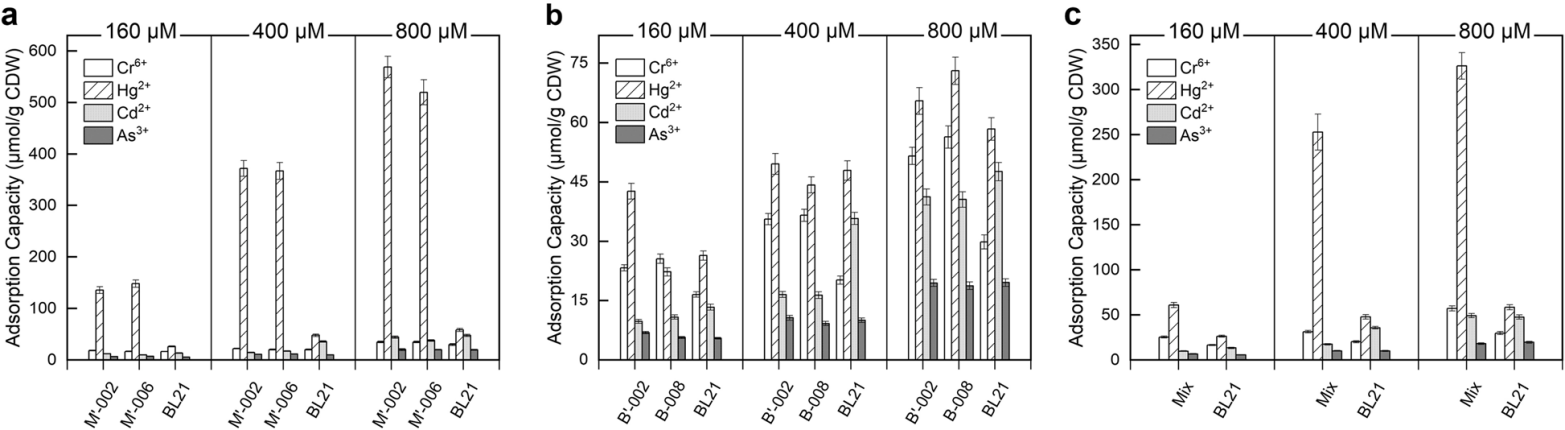
Adsorption performance of engineered strains in mixed metal ion solutions containing Cr^6+^, Hg^2+^, Cd^2+^, and As^3+^ at different concentrations. **a** Mercury adsorption strains; **b** Chromium adsorption strains; **c** Mixture of strains M’-002 and B-008. The mixed metal ion concentrations were set to 160, 400, and 800 μM, respectively. All data shown are the mean ± SEM of three independent experiments

To confirm whether the engineered strains capable to adsorb heavy metals in mixed ion solutions, different concentrations of mixed ions, including Hg^2+^, Cr^6+^, As^3+^, and Cd^2+^, were added to the cultured bacteria. The adsorption capacity of the four engineered strains in mixed-ion solution is shown in Fig. 4a and b. All of the heavy metal adsorption strains performed at significantly higher levels in adsorbing corresponding Hg^2+^/Cr^6+^ than the parental strain in line with the intended purpose, while maintaining the same adsorption capacity to other metals. This is evidence of the specificity of improvement on adsorption performance for the resulting construction. Since the high affinity to Hg^2+^ of BL21 itself, B’-002 and B-008 achieved comparable or even higher Hg^2+^ adsorption capacity than Cr^6+^ adsorption.

#### Comparison of removal rates

**Fig. 5:**
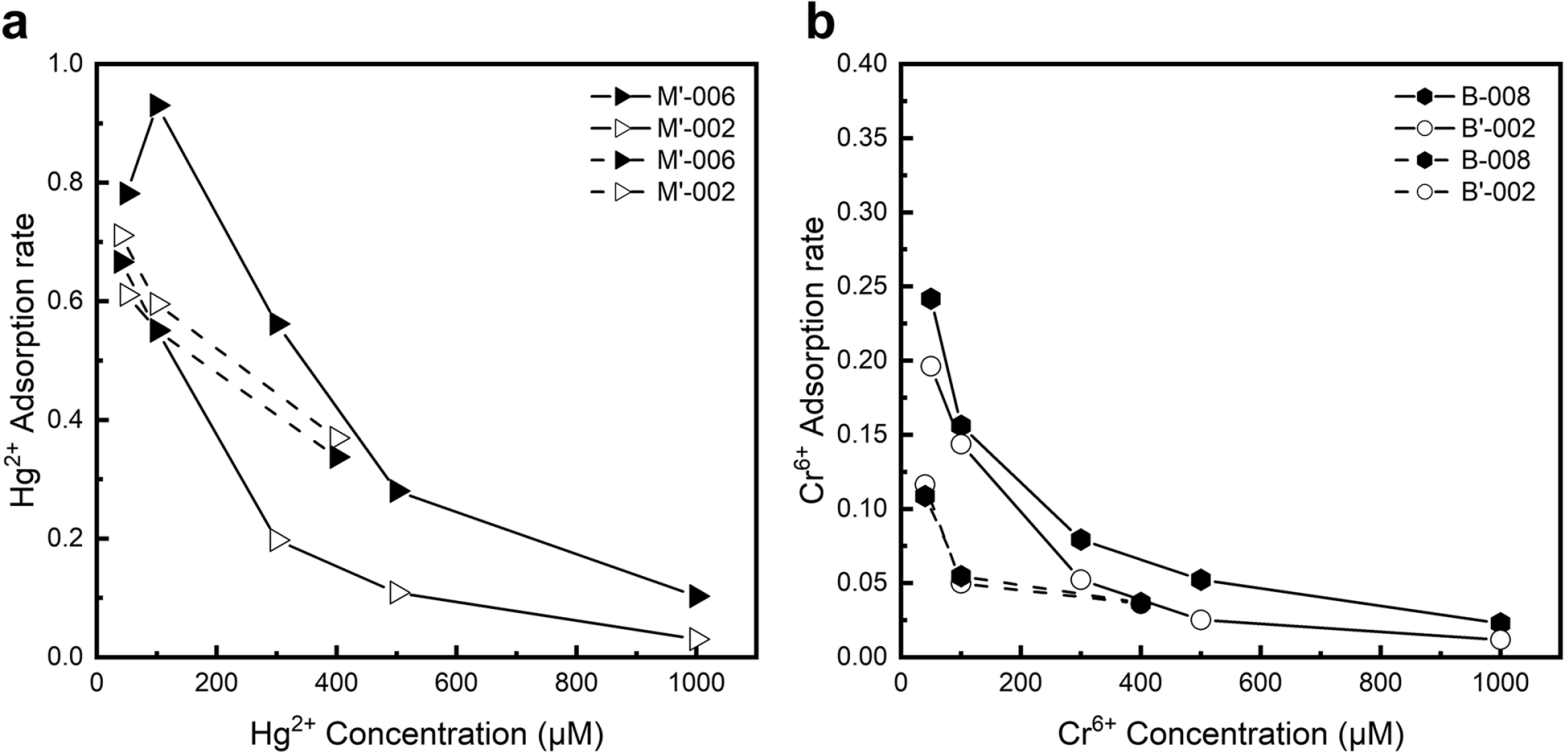
Comparison of the adsorption rates of four engineered strains in the adsorption of single and mixed ions. The solid lines indicate adsorption of single ions, and the dashed lines indicate adsorption of mixed ions. **a** The mercury ion adsorption rates of M'-002 and M'-006 in single and mixed ion solutions; **b** The chromium ion adsorption rates of B'-002 and B-008 in single and mixed ion solutions

The removal rates of single and mixed ions by the adsorption strains at different concentrations were calculated and compared. As shown in Fig. 5, due to the excellent adsorption of Hg^2+^ by MerR, both M’-002 and M’-006 were able to achieve high removal rates (> 50%) at low Hg^2+^ concentrations, both in single- and mixed-ion solutions.

However, the absorption rates sharply dropped as the concentration increased. This indicates that the cells reached saturation and absorption capacity became relatively limited at higher metal concentrations, and that these absorption strains are more suitable for lower concentrations solutions. The chromium adsorption strain started with a very low removal rate (< 30%), which decreased further with increasing ion concentrations. Although the same construction method was used, the inefficiency of ChrB itself limited the ability of the chromium adsorption strains, and the improvement of their adsorption effect will be a direction of subsequent research.

### Performance of a mixed bacterial system

Although there have been many studies on sorbents for bioremediations, most experiments have targeted only one pollutant, while real sewage samples are usually a mixture of multiple pollutants. Therefore, it is more relevant to develop adsorption systems that remove multiple pollutants at once. We mixed and cultured the two strains M’-002 and B-008, which had the highest adsorption performance for Hg^2+^ and Cr^6+^ in this study, under their optimal induction conditions for heavy metal adsorption capacity, and investigated the effect of the mixed bacterial system on the heavy metal adsorption capacity.

The adsorption capacity of the mixed bacterial system for a single ion was firstly examined. As shown in Fig. 3c and d, consistent with the trend of adsorption by single bacterial strains, the adsorption of both Hg^2+^ and Cr^6+^ by the mixed bacteria elevated to a peak and then dropped with the increasing ion concentration. When the ion concentration increased to 500 μM, the adsorption capacities of the mixed bacteria for Hg^2+^ and Cr^6+^ reached a maximum of 449.04 and 50.71 μmol/g CDW, respectively. However, the adsorption decreased with a further increase in heavy metal concentration, which implied that the mixed bacterial system was still affected by the toxicity of the ions in a similar way. The adsorption of both ions by the mixed bacteria exceeded that of wild-type BL21, demonstrating that the modification and optimization of the bacterium were effective. Compared with the single strain adsorption, mixed strains achieve the similar Cr^6+^ adsorption but lower Hg^2+^ adsorption under most concentration. The most likely reason for this reduced ability to adsorb mercury is that these strains lack the necessary genes needed for efficient handling of and tolerance to mercury. HMBPs are elements of heavy metal detoxicity system, the strains lack of MerR/ChrB can be very susceptible to the effects of the heavy metal ions. In the mixed bacterial system, the strains without MerR or ChrB co-exist in the culture. They are less able to withstand the effects of the nonspecific heavy metal ions, therefore interfered in both metal ion solution. As mentioned before, mercury is more toxic than chromium, meaning the toxicity of mercury was having more of a negative impact on the adsorption capability of the chromium adsorption strains., this can be an explanation of why mixed strains performed worse only in Hg^2+^ measurement.

In the study of mixed ion adsorption, the adsorption capacity of both groups increased with the concentration of mixed metal ions. As shown in Fig. 4c, both strains had the highest adsorption capacity for Hg^2+^, which was significantly higher in the mixed bacteria than in wild-type BL21. Although the adsorption capacity of the mixed bacteria for Cr^6+^ was not very high, it was also higher than that of BL21. By contrast, the difference between the adsorption capacity of the mixed bacteria and BL21 for Cd^2+^ and As^3+^ was not significant.

## Discussion

With the increasing pollution of heavy metals in the environment, the biosorption of heavy metals by bacteria has attracted much attention due to its safety, efficiency and practicality, and many studies have been conducted on the use of heavy metal adsorption proteins for the removal of heavy metal ions. In this study, we used HMBPs in combination with different strategies to design different engineered strains, which could adsorb Cr^6+^ and Hg^2+^, respectively. Finally, we obtained four engineered strains (M’-002, M’-006, B’-002, B-008) with better performance through screening.

In order to achieve the highest adsorption effect of the engineered strains, we analyzed the factors affecting HBMPs expression. Firstly, keeping inducer concentration appropriate is necessary to maintain a maximum HMBP concentration within overexpression limits. Since the HMBPs bind metals in proportion of concentration, the adsorption capacity is expected to step with increasing soluble protein expression levels. However, excessive overexpression can lead to cellular stress, and also result protein misfolding and the aggregation of inclusion bodies, which may undermine the function of the HMBPs [46]. Moreover, the expression of heterologous proteins often reaches an upper limit due to the finite recourses in the cell. Therefore, an optimal inducer concentration is expected to give the maximum adsorption capacity, which reaches a balance among induction efficiency, toxicity, and overexpression. Besides, temperature is another important factor that directly affects protein expression. A temperature balance point between maximum protein expression and minimum inclusion bodies is preferred. Higher temperature may push up the protein synthesis rate and accelerate the folding of aggregation intermediates, but the expressed protein is likely to form inclusion bodies. When protein expression is induced at lower temperatures overnight, the proportion of soluble protein will be relatively large in most cases. Eventually, we determined that the optimal inducer concentration in the experiment was 1.0 mM, the induction time was 22 h, and the induction temperature varied with strains.

And then we studied the adsorption capacity of the engineered strains. Past studies on cell adsorption usually consider adsorption to follow The Langmuir and the Freundlich type models [49]. However, biosorption is a complex process based on various mechanisms, including absorption, adsorption, ion exchange, surface complexation, and precipitation [50]. An overly simplified model may not sufficiently capture the complexities of bioengineered modified bacteria. In general, the growing concentration of adsorbent strengthens the driving force of mass transfer, which leads to an enhance in the adsorption capacity of the adsorbent [51]. However, we found that the adsorption capability of each strain under high Hg^2+^/Cr^6+^ concentration declines, which is rarely found in period researches [52–55]. One possible explanation is that as the concentration of Hg^2+^/Cr^6+^ went up, their toxicity was not negligible. High concentrations of heavy metals could become a restriction on HMBP that denatured and limited it continue adsorbing, thereby significantly reducing the adsorption capacity of the bacteria. This could be addressed by enlarging bacterial biomass used for bioremediation or using a multistage remediation process. For surface display strains, a higher copy number of the fusion proteins on the bacterial surface is also an option, but it is limited by the available cell surface area and finite supply of energy/material within the cell. In future research, we plan to determine the optimal gene copy number to achieve a balance between absorbance performance and metabolic burden.

Whether the concentration of heavy metal ions in the samples after adsorption treatment by the engineered strains can reach the standard is also important factor for evaluating the adsorption strains. The Guidelines for Drinking-water Quality (GDWQ) published by WHO provides a guideline value for each pollutant, including heavy metals in drinking water [51]. The recommended levels of Hg^2+^ and Cr^6+^ are 50 and 6 μg per liter of drinking water, respectively, which corresponds to 0.249 μM and 0.115 μM in terms of molar concentrations. This research is pursuing to remove pollutants using adsorption strains in order to bring the effluent water quality up to drinking water standards. From the perspective of drinking water quality standards, the desired target removal rates at different concentrations were calculated and compared with the removal rates of single and mixed ions by the adsorption strains. However, in this study, even M’-006 which had the best adsorption performance failed to meet the requirements of GDWQ. An important reason is that there is an upper limit to the adsorption capacity of the bacterial solution as the adsorption capacity of individual cells is saturated. At low ion concentrations, fewer metal ions can be adsorbed after the same time because there are fewer effective contacts between ions and bacteria. Therefore, two targeted strategies are proposed to achieve water quality standards, i.e., increasing the number of bacteria and extending the adsorption time. Meanwhile, there are often multiple metal ions in the actual process. Because there may be interactions such as competition or synergism between them and the target metal ions, the influence of different metal ions on the adsorption system needs further research [48, 53]. In subsequent studies, we also can try to construct the co-expression stain of protein ChrB and MerR, in order to improve the absorption rate and reduce the cultivation steps in practical applications.

In addition, there are many issues to consider when applying the engineered strains to actual environments. Firstly, both wild strains and complex polluted conditions may greatly affect the engineered strains during the adsorption process. Besides, there is potential environmental impact of the diffusion of engineered strains. Consequently, we intend to immobilize the engineered strains to avoid their diffusion and ensure the adsorption effect [54]. Furthermore, immobilization can enhance strain stability, making adsorption strains robust enough to be desorbed and reused, and the immobilized materials may also increase adsorption of heavy metals [55]. Besides, the separation and management of the biosorption materials require optimization, since the present methods simply treat them as the hazardous waste [51, 56]. We will continue to explore methods of optimization to improve the performance of adsorption strains and achieve our goals.

## Conclusions

In this study, adsorption strains overexpressing MerR or ChrB were constructed to realize heavy metal bioremediation. The adsorption capacity of the engineered strains was efficiently enhanced compared with the wild-type BL21. Under the optimal induction conditions, the adsorption capacity of the engineered bacteria increased and then decreased with increasing of the heavy metal concentration. Notably, the highest Hg^2+^ adsorption capacity reached 658.66 μmol/g CDW, which is the highest value reported to date. Further analyses showed that the performance in mixed-ion solutions was close to that of single-ion solutions, indicating the potential application of the engineered strains under complex conditions.

### Supplementary Information

**Additional file 1: Table S1.** The sources of genes used in this study. **Table S2.** Cell dry weight (g) of the engineered strains at different Hg^2+^/Cr^6+^ concentrations. **Figure S1.** Recombinant plasmid PCR verification gel. **Figure S2.** Growth curves of engineered strains.

## Data Availability

All data generated or analyzed during this study are included in this article and its supplementary information.

## Abbreviations

HMBP: Heavy metal-binding proteins
PCR: Polymerase chain reaction
FL: Flexible linker
RL: Rigid linker
HL: Rigid helical linker
AAS: Atomic absorption spectroscopy
CDW: Cell dry weight
IPTG: Isopropyl‐β‐D‐thiogalactopyranoside
TF: Transcription factor

## References

[1] Khan MU, Malik RN, Muhammad S (2013). Human health risk from heavy metal via food crops consumption with wastewater irrigation practices in Pakistan. Chemosphere.

[2] Tchounwou PB, Yedjou CG, Patlolla AK, Sutton DJ (2012). Heavy metal toxicity and the environment. Exp Suppl.

[3] Kumar A, Singh N, Pandey R, Gupta VK, Sharma B, Rai M, Ingle AP, Medici S (2018). Biochemical and molecular targets of heavy metals and their actions. Biomedical applications of metals.

[4] Chen Q, Yao Y, Li X, Lu J, Zhou J, Huang Z (2018). Comparison of heavy metal removals from aqueous solutions by chemical precipitation and characteristics of precipitates. J Water Process Eng.

[5] Peng H, Guo J (2020). Removal of chromium from wastewater by membrane filtration, chemical precipitation, ion exchange, adsorption electrocoagulation, electrochemical reduction, electrodialysis, electrodeionization, photocatalysis and nanotechnology: a review. Environ Chem Lett.

[6] Jain CK, Ali I (2000). Arsenic: occurrence, toxicity and speciation techniques. Water Res.

[7] Lu X, Wu J, Guo Y (2019). Advances in remediation of heavy metal contaminated soil by biochar. Appl Chem Industry.

[8] Li Y, Yang LM, Xu Z, Sun Q (2017). Separation and recovery of heavy metals from waste water using synergistic solvent extraction. 1st International Conference on New Material and Chemical Industry (NMCI): Nov 19–21 2016; Sanya, Peoples R China.

[9] AlOthman ZA, Alam MM, Naushad M (2013). Heavy toxic metal ion exchange kinetics: validation of ion exchange process on composite cation exchanger nylon 6,6 Zr(IV) phosphate. J Ind Eng Chem.

[10] Castro-Munoz R, Gonzalez-Melgoza LL, Garcia-Depraect O (2021). Ongoing progress on novel nanocomposite membranes for the separation of heavy metals from contaminated water. Chemosphere.

[11] Yin Z, Zhang J, Liao S, Ma Q, Wang Q, Zhang J (2015). Research and application of the remediation technology for the chromium contaminated site. Environ Eng.

[12] Priyadarshanee M, Das S (2021). Biosorption and removal of toxic heavy metals by metal tolerating bacteria for bioremediation of metal contamination: a comprehensive review. J Environ Chem Eng.

[13] Volesky B, Holan ZR (1995). Biosorption of heavy metals. Biotechnol Prog.

[14] Peng WH, Li XM, Xiao ST, Fan WH (2018). Review of remediation technologies for sediments contaminated by heavy metals. J Soils Sediments.

[15] Shao HB, Chu LY, Ruan CJ, Li H, Guo DG, Li WX (2010). Understanding molecular mechanisms for improving phytoremediation of heavy metal-contaminated soils. Crit Rev Biotechnol.

[16] Deniz F, Karabulut A (2017). Biosorption of heavy metal ions by chemically modified biomass of coastal seaweed community: studies on phycoremediation system modeling and design. Ecol Eng.

[17] Volesky B (1994). Advances in biosorption of metals: selection of biomass types. FEMS Microbiol Rev.

[18] Yu ZS, Han HW, Feng PY, Zhao S, Zhou TY, Kakade A, Kulshrestha S, Majeed S, Li XK (2020). Recent advances in the recovery of metals from waste through biological processes. Biores Technol.

[19] Borrok D, Fein JB, Kulpa CF (2004). Proton and Cd adsorption onto natural bacterial consortia: testing universal adsorption behavior. Geochim Cosmochim Acta.

[20] Say R, Yimaz N, Denizli A (2003). Removal of heavy metal ions using the fungus Penicillium canescens. Adsorpt Sci Technol.

[21] Say R, Yilmaz N, Denizli A (2003). Biosorption of cadmium, lead, mercury, and arsenic ions by the fungus Penicillium purpurogenum. Sep Sci Technol.

[22] Chang S, Wu Z, Sun W, Shu H (2018). The construction of an engineered bacterial strain and its application in accumulating mercury from wastewater. Appl Sci-Basel.

[23] Sinha A, Pant KK, Khare SK (2012). Studies on mercury bioremediation by alginate immobilized mercury tolerant Bacillus cereus cells. Int Biodeterior Biodegrad.

[24] Green-Ruiz C (2006). Mercury(II) removal from aqueous solutions by nonviable Bacillus sp from a tropical estuary. Biores Technol.

[25] De Rossi A, Rigon MR, Zaparoli M, Braido RD, Colla LM, Dotto GL, Piccin JS (2018). Chromium (VI) biosorption by Saccharomyces cerevisiae subjected to chemical and thermal treatments. Environ Sci Pollut Res.

[26] Foroutan R, Mohammadi R, Ramavandi B (2018). Treatment of chromium-laden aqueous solution using CaCl_2_-modified Sargassum oligocystum biomass: characteristics, equilibrium, kinetic, and thermodynamic studies. Korean J Chem Eng.

[27] Prabhakaran DC, Bolanos-Benitez V, Sivry Y, Gelabert A, Riotte J, Subramanian S (2019). Mechanistic studies on the bioremediation of Cr(VI) using Sphingopyxis macrogoltabida SUK2c, a Cr(VI) tolerant bacterial isolate. Biochem Eng J.

[28] Zhou M, Liu Y, Zeng G, Li X, Xu W, Fan T (2007). Kinetic and equilibrium studies of Cr(VI) biosorption by dead Bacillus licheniformis biomass. World J Microbiol Biotechnol.

[29] Saranya K, Sundaramanickam A, Shekhar S, Meena M, Sathishkumar RS, Balasubramanian T (2018). Biosorption of multi-heavy metals by coral associated phosphate solubilising bacteria Cronobacter muytjensii KSCAS2. J Environ Manage.

[30] Pulimi M, Jamwal S, Samuel J, Chandrasekaran N, Mukherjee A (2012). Enhancing the hexavalent chromium bioremediation potential of acinetobacter junii VITSUKMW2 using statistical design experiments. J Microbiol Biotechnol.

[31] Dash HR, Das S (2012). Bioremediation of mercury and the importance of bacterial mer genes. Int Biodeterior Biodegrad.

[32] Huang CC, Narita M, Yamagata T, Itoh Y, Endo G (1999). Structure analysis of a class II transposon encoding the mercury resistance of the gram-positive bacterium Bacillus megaterium MB1, a strain isolated from Minamata Bay, Japan. Gene.

[33] Norambuena J, Miller M, Boyd JM, Barkay T (2020). Expression and regulation of the mer operon in *Thermus thermophilus*. Environ Microbiol.

[34] Barkay T, Miller SM, Summers AO (2003). Bacterial mercury resistance from atoms to ecosystems. FEMS Microbiol Rev.

[35] Huang CC, Narita M, Yamagata T, Endo G, Silver S (2002). Characterization of two regulatory genes of the mercury resistance determinants from TnMERI1 by luciferase-based examination. Gene.

[36] Zeng QD, Stalhandske C, Anderson MC, Scott RA, Summers AO (1998). The core metal-recognition domain of MerR. Biochemistry.

[37] Song LY, Caguiat J, Li ZR, Shokes J, Scott RA, Olliff L, Summers AO (2004). Engineered single-chain, antiparallel, coiled coil mimics the MerR metal binding site. J Bacteriol.

[38] Seneque O, Rousselot-Pailley P, Pujol A, Boturyn D, Crouzy S, Proux O, Manceau A, Lebrun C, Delangle P (2018). Mercury trithiolate binding (HgS3) to a de novo designed cyclic decapeptide with three preoriented cysteine side chains. Inorg Chem.

[39] Ghosh D, Lee KH, Demeler B, Pecoraro VL (2005). Linear free-energy analysis of mercury(II) and cadmium(II) binding to three-stranded coiled coils. Biochemistry.

[40] Branco R, Chung AP, Johnston T, Gurel V, Morais P, Zhitkovich A (2008). The chromate-inducible chrBACF operon from the transposable element TnOtChr confers resistance to chromium(VI) and superoxide. J Bacteriol.

[41] Branco R, Alpoim MC, Morais PV (2004). Ochrobactrum tritici strain 5bvl1 - characterization of a Cr(Vi)-resistant and Cr(Vi)-reducing strain. Can J Microbiol.

[42] Aguilar-Barajas E, Paluscio E, Cervantes C, Rensing C (2008). Expression of chromate resistance genes from *Shewanella sp*. strain ANA-3 in Escherichia coli. FEMS Microbiol Lett.

[43] Branco R, Morais PV (2013). Identification and characterization of the transcriptional regulator ChrB in the chromate resistance determinant of ochrobactrum tritici 5bvl1. PLoS One.

[44] Naguib M, El-Gendy A, Khairalla A (2018). Microbial diversity of mer operon genes and their potential rules in mercury bioremediation and resistance. Open Biotechnol J.

[45] Mergeay M, Van Houdt R (2021). *Cupriavidus metallidurans* CH34, a historical perspective on its discovery, characterization and metal resistance. FEMS Microbiol Ecol.

[46] Nies DH, Rehbein G, Hoffmann T, Baumann C, Grosse C (2006). Paralogs of genes encoding metal resistance proteins in *Cupriavidus metallidurans* strain CH34. J Mol Microbiol Biotechnol.

[47] Park M (2020). Surface display technology for biosensor applications: a review. Sensors.

[48] Sarangi A, Krishnan C (2008). Comparison of in vitro Cr(VI) reduction by CFEs of chromate resistant bacteria isolated from chromate contaminated soil. Biores Technol.

[49] Jia XQ, Li Y, Xu T, Wu K (2020). Display of lead-binding proteins onEscherichia colisurface for lead bioremediation. Biotechnol Bioeng.

[50] Brown NL, Stoyanov JV, Kidd SP, Hobman JL (2003). The MerR family of transcriptional regulators. FEMS Microbiol Rev.

[51] Beni AA, Esmaeili A (2020). Biosorption, an efficient method for removing heavy metals from industrial effluents: a review. Environ Technol Innov.

[52] Pal A, Bhattacharjee S, Saha J, Sarkar M, Mandal P (2022). Bacterial survival strategies and responses under heavy metal stress: a comprehensive overview. Crit Rev Microbiol.

[53] Li H, Huang S, Zhang Y (2016). Cr(VI) removal from aqueous solution by thermophilic denitrifying bacterium *Chelatococcus daeguensis* TAD1 in the presence of single and multiple heavy metals. J Microbiol.

[54] McCarthy D, Edwards GC, Gustin MS, Care A, Miller MB, Sunna A (2017). An innovative approach to bioremediation of mercury contaminated soils from industrial mining operations. Chemosphere.

[55] Dash HR, Das S (2015). Bioremediation of inorganic mercury through volatilization and biosorption by transgenic *Bacillus cereus* BW-03(pPW-05). Int Biodeterior Biodegrad.

[56] Xin B, Wang J (2022). Scientific definition of hazardous wastes containing heavy metals and their resource utilization. Chin J Environ Eng.

