# Supplementary material for: Adsorption of Hg2+/Cr6+ by metal-binding proteins heterologously expressed in Escherichia coli
Source: BMC Biotechnol. 2024 Mar 23;24:15. doi: 10.1186/s12896-024-00842-9 (PMC10960487; doi:10.1186/s12896-024-00842-9)
Supplement: Supplementary file 1 — Additional file 1: Table S1. The sources of genes used in this study. Table S2. Cell dry weight (g) of the engineered strains at different Hg2+/Cr6+ concentrations. Figure S1. Recombinant plasmid PCR verification gel. Figure S2. Growth curves of engineered strains. [file 12896_2024_842_MOESM1_ESM.docx]

## Supplementary Materials

**Table S1** Primers used in this study

| **Primer** | **Sequence（5’-3’）** | **Restriction site** |
| --- | --- | --- |
| HindIII-INPN | CCCAAGCTTATGACTCTCGACAAGGC | HindIII |
| NheI-INPN | CTAGCTAGCGGTCTGCAAATTCTGCG | NheI |
| NheI-FL | CTAGCTAGCGGAACCACCACCACCGG | NheI |
| NheI-HL | CTAGCTAGCAGCCTTAGCAGCAGCCTCC | NheI |
| NheI-RL | CTAGCTAGCTGGAGCTGGAGCTGGGG | NheI |
| NheI-96 bp | CTAGCTAGCAATTAGATCACTGTGGTTG | NheI |
| NheI-ChrB | CTAGCTAGCATGAACGCTCTCCCATCCTCTCCCGAGACTGCCTG | NheI |
| NheI-ChrB_opt_ | CTAGCTAGCATGAATGCACTGCCGAGCAGTCCGGAAACCGCATG | NheI |
| NheI-MerR | CTAGCTAGCATGGAAAACAATTTGGAGAACCTGACCATTGGCG | NheI |
| NheI-MerR_opt_ | CTAGCTAGCATGGAAAATAACCTGGAAAACCTGACGATTGGTG | NheI |
| XhoI-ChrB | CCGCTCGAGTCAGTGGTGATGGTGATGATGGCGTTTGCGCGGG | XhoI |
| XhoI-ChrB_opt_ | CCGCTCGAGTCAGTGGTGATGGTGATGATGGCGTTTACGCGGAC | XhoI |
| XhoI-MerR | CCGCTCGAGTCAGTGGTGATGGTGATGATGAGGCATAGCCGAAC | XhoI |
| XhoI-MerR_opt_ | CCGCTCGAGTCAGTGGTGATGGTGATGATGCGGCATAGCAGAAC | XhoI |
| 28a-F1 | ATTCGAGCTCCGTCGAC |  |
| 28a-R1 | TGGTGGTGGTGGTGGTG |  |
| BamHI-ChrB | CGCGGATCCTCAGCGTTTACGCGGAC | BamHI |
| BamHI-ChrB_opt_ | CGCGGATCCCTAGCGTTTGCGCGGGC | BamHI |
| BamHI-MerR | CGCGGATCCCTAAGGCATAGCCGAAC | BamHI |
| BamHI-MerR_opt_ | CGCGGATCCTTACGGCATAGCAGAAC | BamHI |
| EcoRI-ChrB | CCGGAATTCTCAGCGTTTACGCGGAC | EcoRI |
| EcoRI-ChrB_opt_ | CCGGAATTCCTAGCGTTTGCGCGGGC | EcoRI |
| EcoRI-MerR | CCGGAATTCCTAAGGCATAGCCGAAC | EcoRI |
| EcoRI-MerR_opt_ | CCGGAATTCTTACGGCATAGCAGAAC | EcoRI |
| 28a-F2 | ATTCGAGCTCCGTCGAC |  |
| 28a-R2 | TGGTGGTGGTGGTGGTG |  |

**Table S2** Cell dry weight (g) of the engineered strains at different Hg^2+^/Cr^6+^ concentrations. All cell dry weights were subtracted from the adsorbed heavy metal. The weights of heavy metals were calculated by moles and molar mass.

| **Strain** | **Hg^2+^/Cr^6+^ concentration (μM)** | | | | |
| --- | --- | --- | --- | --- | --- |
|  | 50 | 100 | 300 | 500 | 1000 |
| M’-002 | 0.017 | 0.015 | 0.008 | 0.008 | 0.006 |
| M’-006 | 0.030 | 0.030 | 0.025 | 0.025 | 0.025 |
| B’-002 | 0.063 | 0.057 | 0.055 | 0.053 | 0.053 |
| B-008 | 0.064 | 0.061 | 0.057 | 0.056 | 0.053 |


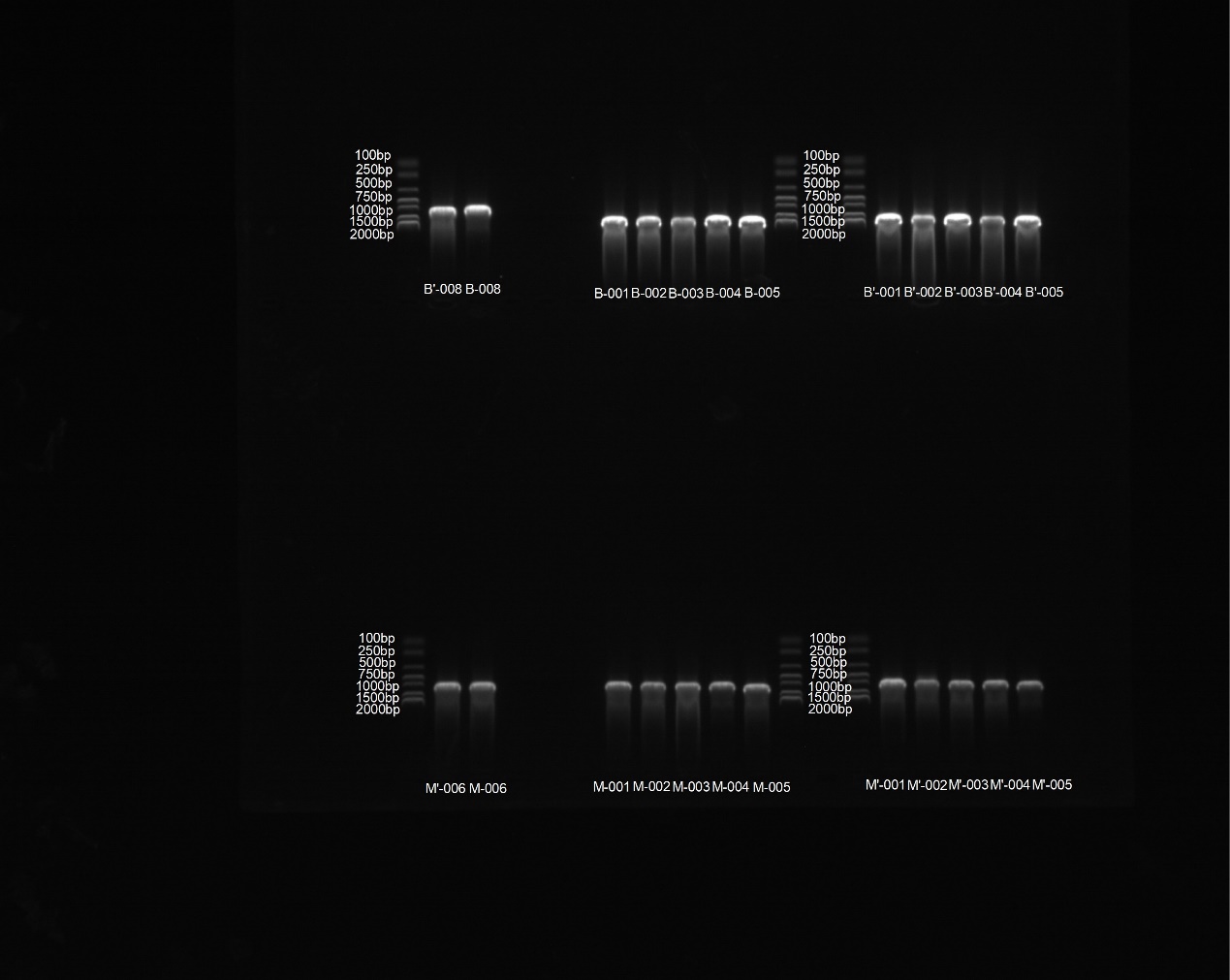


**Figure S1** Recombinant plasmid PCR validation gel shows all bands in the correct position.





**Figure S2** Growth curves of engineered strains. Like the plasmid-free BL21, all engineered strains kept growing for the first 16 hours of incubation, stopped growing after 16 h, and maintained an OD_600_ value of about 3.0. (a) and (b): Intracellular adsorption strains; (c)~(f): Extracellular adsorption strains.
